# The Diverse Roles of TAO Kinases in Health and Diseases

**DOI:** 10.3390/ijms21207463

**Published:** 2020-10-10

**Authors:** Chih-Yeu Fang, Tsung-Ching Lai, Michael Hsiao, Yu-Chan Chang

**Affiliations:** 1 Genomics Research Center, Academia Sinica, Taipei 115, Taiwan; phildts@gmail.com; 2 Division of Pulmonary Medicine, Department of Internal Medicine, Wan Fang Hospital, Taipei Medical University, Taipei 116, Taiwan; chuching0305@gmail.com; 3 Pulmonary Research Center, Wan Fang Hospital, Taipei Medical University, Taipei 116, Taiwan; 4 Department of Biochemistry, College of Medicine, Kaohsiung Medical University, Kaohsiung 807, Taiwan; 5 Department of Biomedical Imaging and Radiological Science, National Yang-Ming University, Taipei 112, Taiwan

**Keywords:** thousand and one kinase, p38 MAPK, SAPK/JNK, Hippo, TAOK

## Abstract

Thousand and one kinases (TAOKs) are members of the MAP kinase kinase kinase (MAP3K) family. Three members of this subfamily, TAOK1, 2, and 3, have been identified in mammals. It has been shown that TAOK1, 2 and 3 regulate the p38 MAPK and Hippo signaling pathways, while TAOK 1 and 2 modulate the SAPK/JNK cascade. Furthermore, TAOKs are involved in additional interactions with other cellular proteins and all of these pathways modulate vital physiological and pathophysiological responses in cells and tissues. Dysregulation of TAOK-related pathways is implicated in the development of diseases including inflammatory and immune disorders, cancer and drug resistance, and autism and Alzheimer’s diseases. This review collates current knowledge concerning the roles of TAOKs in protein–protein interaction, signal transduction, physiological regulation, and pathogenesis and summarizes the recent development of TAOK-specific inhibitors that have the potential to ameliorate TAOKs’ effects in pathological situations.

## 1. Introduction

Kinases are enzymes that catalyze the transfer of phosphate groups from a phosphate-donating molecule (such as ATP) to their substrate molecule, which can be a protein, lipid, or carbohydrate. The phosphorylation state of a substrate can affect its activity and ability to bind to and regulate other molecules. Mitogen-activated protein kinases (MAPKs) phosphorylate serine/threonine residues of their substrates, resulting in activation or de-activation of the downstream targets [1]. MAP kinases are evolutionarily conserved and ubiquitously expressed in eukaryotes. The sterile 20 (STE20) kinase of *Saccharomyces cerevisiae* functions downstream of a heterotrimeric G protein but upstream of MAP kinases in the MAPK cascade of the yeast pheromone response pathway [2,3]. While searching for the STE20 kinase ortholog in mammals, the TAO kinases were identified. The first “thousand and one” kinase, TAOK1, was identified in the rat by fishing the cDNA library with a degenerate STE20 kinase probe [4]. It is named “thousand and one” because 1001 amino acids are encoded by the TAOK1 gene. A second TAO kinase with closely related sequences, TAOK2, was also identified and later characterized [4,5]. The third TAO kinase, human TAOK3, was identified by its association with EPS8 (EGFR kinase substrate 8) protein in an expression library assay [6]. Human TAO kinase orthologs (TAOK1, TAOK2, and TAOK3) were identified subsequently [7,8]. TAOKs are involved in various cellular signaling pathways including the p38/MAPK14 stress-activated MAPK cascade, the JNK/SAPK cascade, and the Salvador–Warts–Hippo cascade. In addition, TAOKs are found to interact with other cellular proteins both dependent and independent of their kinase activity. Through these interactions, TAOKs regulate the DNA damage responses, cytoskeleton stability, apoptosis, and other physiological and pathophysiological responses.

MAPK pathways participate in the transduction of extracellular changes into coordinated and integrated intracellular adaptive responses. MAPKs respond to diverse stimuli, including mitogens, osmotic stress, heat shock, and inflammatory cytokines, and regulate vital cellular processes such as mitosis, proliferation, differentiation, apoptosis, stress, and immune responses [9]. Activation of a MAPK cascade occurs via the multi-tiered, consecutive phosphorylation of downstream targets. When triggered by stimuli, the first kinase in the cascade, the MAP3K (MAP kinase kinase kinase) is phosphorylated and activated by protein kinases downstream of surface G protein-coupled receptors [10]. In a classical MAPK cascade, the MAP3K then activates a downstream MAP2K (MAP kinase kinase or MKK), which, in turn, activates a MAPK [9]. Upon activation, the MAPK phosphorylates various targets in the cytosol and nucleus to alter protein function or gene expression appropriate biological responses. Three classical MAPK pathways are known in mammalian cells: the extracellular signal-regulated kinase 1/2 (ERK1/2), the p38 MAPK, and the c-JUN N-terminal kinase (JNK) pathways. ERK1/2 is activated in response primarily to hormones, growth factors, and proinflammatory stimuli, while p38 MAPKs and JNKs respond to cellular and environmental stresses, such as inflammatory cytokines, oxidative stress, DNA damage, ultraviolet irradiation, heat, and osmotic shock. Among these three classical MAPK pathways, TAOKs are currently known to be involved in the regulation of the p38 MAPK and JNK cascades.

The Hippo signaling pathway, also known as the Salvador–Warts–Hippo (SWH) pathway, controls organ size through regulation of cell proliferation and apoptosis [11]. This pathway also regulates the self-renewal and expansion of stem cell and tissue-specific progenitor cells. The Hippo pathway is also a kinase cascade, wherein a series of activated kinases phosphorylate the transcription co-activators YAP (Yes-associated protein) and TAZ (transcriptional coactivator with PDZ-binding motif) and inhibit their nuclear translocation. Lack of the transcriptional activator YAP/TAZ results in the downregulation of genes that support cell proliferation and inhibit apoptosis. Given that the Hippo cascade is involved in controlling cell proliferation and modulating apoptosis, dysfunction of this pathway could play an important role in the development of human cancer [12]. TAOKs have also been found to be regulators of the Hippo signaling pathway, in addition to their involvement in the MAPK cascades.

In this review, we summarize current knowledge concerning the signaling pathways regulated by TAOKs and their correlation to physiological regulation and disease progression. Finally, we discuss the progress toward the development of a selective and potent TAOK inhibitor, which may be of benefit in combating TAOK-associated pathogenesis.

## 2. Structure and Function of TAO Kinases

**Figure 1:**
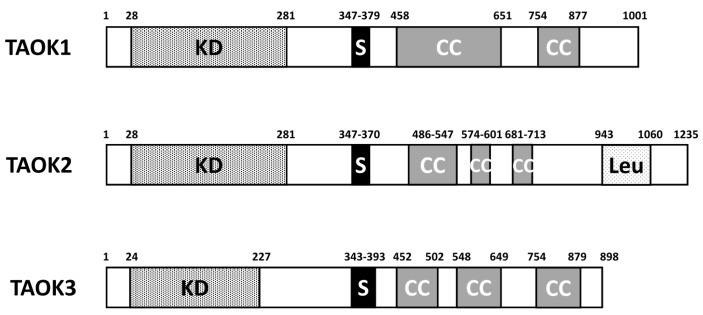
Diagram of domain structure of human TAOK1/2/3. KD: kinase domain; S: serine-rich domain; CC: coiled coil regions; Leu: leucine-rich repeat.

There are three members in the TAO kinase family: TAOK1, TAOK2, and TAOK3. In humans, TAOK1 is located on chromosome 17p at position 11.2, while TAOK2 is on 16p11.2 and TAOK3 is on 12q24.23. Several splice variants are noted for all three TAOKs. They share similar domain structures despite the difference in the amino acid length. TAOKs are serine/threonine-protein kinases noted for their N-terminal positioning of the kinase domain (Figure 1). A serine-rich domain is located around 350–380 a. a. Two to three coiled coil regions are located in the C-terminal half of TAOKs. In TAOK2, there is a leucine-rich repeat situated close to the C-terminal end. The human TAOK2 protein kinase domain displays 89.8% amino acid identity to TAOK1 [5], while the homology of the kinase domain of TAOK3 to TAOK1 and TAOK2 is 88.6% and 82.7%, respectively [8]. Generally, TAOKs are ubiquitously expressed in most tissues, with the highest levels in the testes and brain [6,8]. One exception is that TAOK3 is highly expressed in peripheral blood leukocytes, spleen, and thymus, while TAOK1 and TAOK2 are low in these tissues. The high expression of TAOK1/2 in the brain and TAOK3 in myeloid/lymphoid tissues may reflect the tissue-specific functions of individual TAOKs.

## 3. Signaling Pathways and Cellular Physiologies Regulate by TAO Kinases

TAOKs have been reported to regulate the p38/MAPK, JNK/SAPK, and Hippo signaling pathways. In addition to these signaling pathways, TAOKs also interact with other proteins and regulate several additional cellular physiological functions. These findings are highlighted in the following sections.

### 3.1. TAO Kinases Regulate the p38/MAPK Pathway

p38 MAPKs are activated by stress stimuli and involved in cell differentiation, apoptosis, and autophagy. Four members of this family, p38 MAPK α (MAPK14), β (MAPK11), δ (MAPK12/ERK6), and γ (MAPK13/SAPK4), have been identified. As with typical MAPK cascades, the first component is a MAP3K that phosphorylates and activates MKK3/6 (the MAP2Ks), and MKK3/6 then phosphorylates and activates the p38 MAPK. p38 MAPK is involved in the regulation of several cytosolic cofactors and nuclear transcription factors.

**Figure 2:**
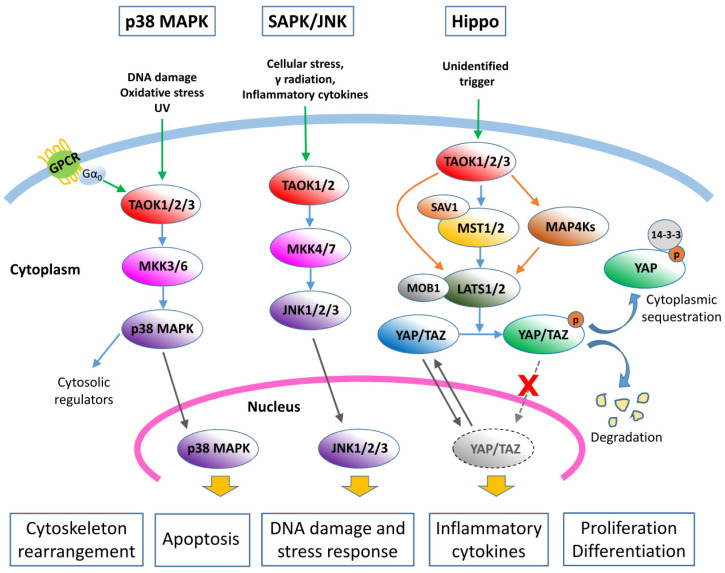
Kinase cascades regulated by TAOK1/2/3. TAO kinases are involved in p38 MAPK, SAPK/JNK, and Hippo signaling pathways. GPCR: G protein-coupled receptor; SAV1: Salvador 1. Green arrows: upstream stimuli; blue arrows: typical/canonical pathways; orange arrows: alternative pathways in the Hippo cascades; grey arrows: nuclear translocation/exportation.

TAOKs, through their activity as MAP3Ks, were found to activate the p38 MAPK pathways (Figure 2) [4,5,8]. TAOKs are activated intensely by ionizing and ultraviolet radiation, indicating a primary function in response to DNA damage via p38 activation. Under these conditions, Raman et al. [13] reported that TAOKs are activated by ATM (ataxia telangiectasia mutated) phosphorylation to regulate p38-mediated DNA damage responses. In addition, the heterotrimeric G protein Gα_0_ was also found to activate TAOK2 and the downstream p38 cascades [14]. It has therefore been suggested that TAOKs are the intermediates that link specific G protein-coupled receptors (GPCRs) to the p38 MAPK pathway (Figure 2) [14]. Activated TAOKs phosphorylate MKK3/6, which then phosphorylate p38 kinases [4,5,15] (Figure 2). Chen et al. [5,15] confirmed that TAOK2 docks to MKK3 through a region (314–451 a.a.) adjacent to the TAOK kinase domain. Taken together, these findings reveal that TAOKs play the intermediate MAP3K roles that link environmental stimuli to the p38 MAPK signaling pathway (Figure 2).

### 3.2. TAO Kinases Regulate the SAPK/JNK Pathway

Stress-activated protein kinases (SAPK)/c-Jun N-terminal kinases (JNK) belong to the MAPK family and are activated by a variety of environmental stresses. JNK1, 2, and 3 respond to stimuli including cytokines, ultraviolet irradiation, heat, and osmotic shock. Their activation is carried out by two MAP2Ks, MKK4 and MKK7, which are activated by upstream MAP3Ks. Activated JNK translocates to the nucleus, where it regulates the activity of multiple transcription factors [16]. JNKs are involved in cell proliferation, differentiation, apoptosis, neurodegeneration, and inflammatory responses [17].

In addition to MKK3/6 in the p38 MAPK cascade, TAOK1 and TAOK2 were found to phosphorylate MKK4/MKK7 and activate the JNK signaling cascade (Figure 2) [5,7,18,19]. Treatment with the apoptosis-inducing agents paclitaxel and staurosporine activated endogenous TAOK 1 and 2 and JNK pathways [18,19]. Overexpression of TAOK2 also activated the endogenous JNK/SAPK cascade in HEK293 cells [15]. The involvement of TAOK3 in the SAPK/JNK pathway is somewhat controversial. While an initial study by Tassi et al. [6] showed that TAOK3 inhibits the basal activity of SAPK/JNK and diminishes its activation in response to human epidermal growth factor in COS7 cells, a later report by Zhang et al. [20] indicated that TAOK3 activated SAPK/JNK when transfected in NIH3T3 cells. A more recent study by MacKeigan et al. [21] demonstrated that downregulation of TAOK3 resulted in rapid activation of JNK1/2 and caspase-9, and PARP cleavage, which led to apoptosis in HeLa cells, whilst a study by Kapfhamer et al. [22] showed that the level of activated phosphor-JNK was higher in the brain of the TAOK3-disrupted mouse as compared to the control mouse, which suggests that TAOK3 is a negative regulator of the SAPK/JNK cascade. The inconsistency of JNK activation by TAOK3 could be a consequence of differences in cellular context. Additional studies are required to validate the role of TAOK3 in the JNK pathway. Currently, it is generally believed that TAOK 1 and 2 are activators of the SAPK/JNK pathway while TAOK3 is not.

### 3.3. TAO Kinases Regulate the Hippo Signaling Pathway

The Hippo pathway is also a kinase cascade, wherein MST1/2 kinases (mammalian STE20-like 1/2; the ortholog of Drosophila Hippo) and SAV1 (Salvador 1) form a complex to phosphorylate and activate LATS1/2 (large tumor suppressor 1/2; the ortholog of Drosophila Warts). LATS1/2 sequentially phosphorylate the transcription co-activators YAP and TAZ and inhibit their nuclear translocation by retaining them in the cytosol or targeting them for degradation. Non-phosphorylated YAP/TAZ translocate into the nucleus and interact with TEAD (transcriptional enhanced associate domain) and other transcription factors to induce genes that support cell proliferation and inhibit apoptosis. YAP/TAZ can also reprogram cancer cells into cells with stem-like traits [23]. In this cascade, genes involved in the phosphorylation of YAP/TAZ are identified as tumor suppressors, whereas YAP/TAZ are recognized as oncogenes [24].

Intriguingly, it was found that loss of TAO1 (TAOK1 ortholog in Drosophila) upregulated Hippo signaling targets (Figure 2) [25]. TAO1 was shown to phosphorylate Hippo to activate the pathway, which then functions to restrict cell proliferation in Drosophila [26]. A study of human TAOK1 in 293T cells also showed that TAOK1 induced substantial phosphorylation of the Hippo ortholog MST2 [26]. By using a TAOK1/2/3-knockout model of HEK293A cells, Plouffe et al. [27] showed that TAOKs act not only upstream of MST1/2, but also in parallel to directly activate LATS1/2, and that eliminating TAOKs significantly decreased phosphorylation of YAP/TAZ and their cytoplasmic retention. In addition, Meng et al. [28] found that knockout of MAP4K4, 6, and 7 significantly blocked TAOK1-induced YAP phosphorylation, indicating that TAOK1 may also act through MAP4K4/6/7 to activate LATS1/2. Collectively, TAOKs are regulators of the Hippo signaling pathway and their activation suppresses YAP/TAZ transactivation ability (Figure 2). Although proteins in the Hippo signaling pathway that decrease the YAP/TAZ activity are regarded as potential tumor suppressors [24,26], currently there is no direct evidence indicating that TAOKs play specific roles in tumor suppression via the Hippo cascade.

### 3.4. Cytoskeleton Regulations by TAO Kinases

**Figure 3:**
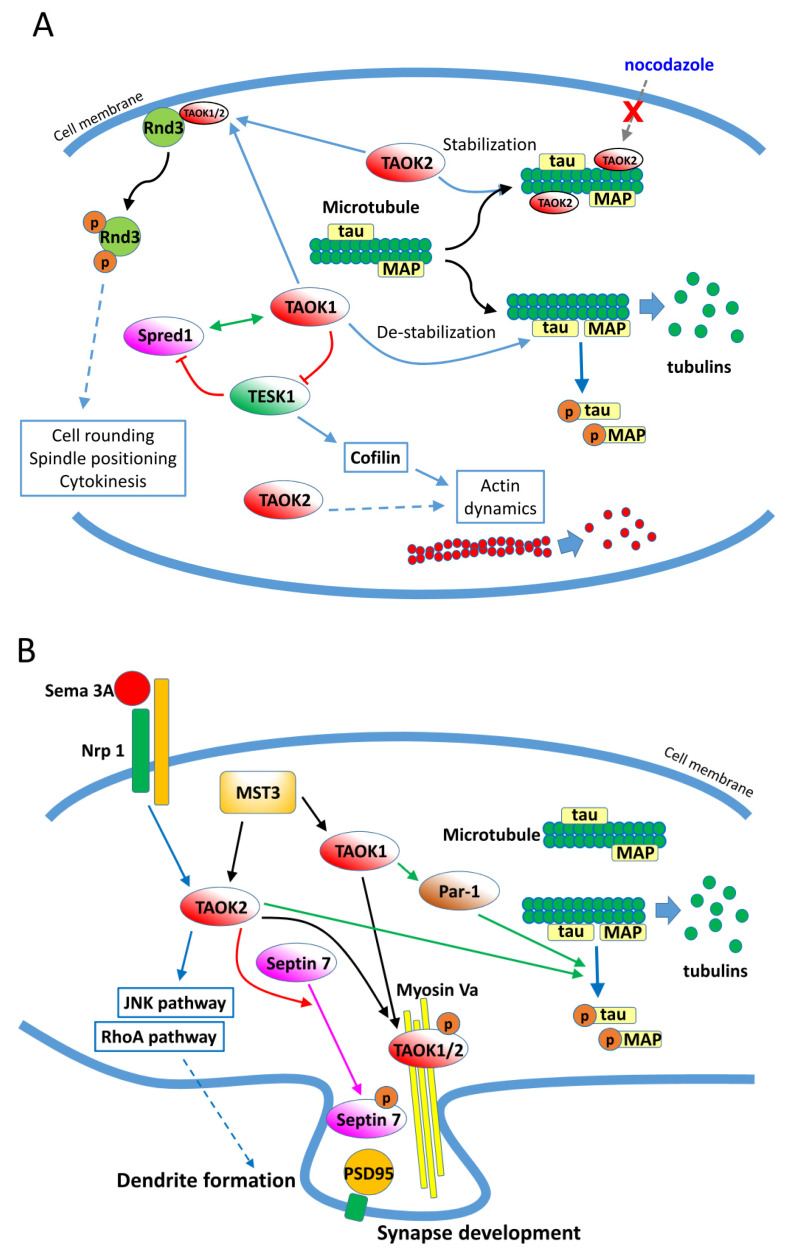
Regulation of cytoskeleton components by TAOKs. (**A**) Regulation of microtubules and actin filaments by TAOK1 and TAOK2 in normal cells. (**B**) Signaling pathways modulated by TAOK1 and TAOK2 in neurons during development and dendrite/synapse formation. MAP: microtubule-binding proteins.

In addition to the aforementioned signaling pathways, TAOKs are reported to interact with other cytosolic proteins and be involved in additional physiological processes. TAOKs modulate the dynamics and organization of several cytoskeleton components [29,30,31]. TAOKs are activated catalytically during mitosis and neuritogenesis [32,33,34,35,36]. By phosphorylation of microtubule-binding proteins including tau, TAOK1 induces microtubule instability by causing dissociation of tau from microtubules, which results in their disassembly [30,31,37] (Figure 3A). Conversely, TAOK2 binds directly to microtubules through its C-terminal region (amino acids 745–1235) and stabilizes microtubules at the perinuclear regions. Furthermore, the TAOK2-stabilized microtubules are resistant to nocodazole-induced depolymerization [29]. TAOK2 was found to increase the levels of acetylated α-tubulin when associated with microtubules and also to bind and phosphorylate α- and β-tubulin in vitro [29]. It was demonstrated that in mitotic cells, activated TAOK1 localizes to the cytoplasm while TAOK2 localizes to the centrosomes, and both TAOKs are required for spindle positioning and mitotic cell rounding [33,35]. Garg et al. [38] further showed that TAOK1 and 2 bind and phosphorylate the atypical Rho family protein Rnd3 and elicit the translocation of Rnd3 from the plasma membrane to the cytosol, which contributes to spindle positioning, mitotic cell rounding, and cytokinesis (Figure 3A). Additionally, TAOKs may regulate the dynamics of actin. Cells overexpressing TAOK2 are found to be rounded, have fewer processes, and show a marked reduction in actin stress fibers. The alteration in cell morphology and actin organization by TAOK2 was dependent on both its C-terminal actin-binding motif and kinase activity [7]. It was also found that TAOK1, Sprouty-related protein with EVH-1 domain1 (Spred1), and testis-specific protein kinase (TESK1) form a three-way interacting network that modulates the dynamics of both microtubules and actin in CHO cells [39] (Figure 3A). In Drosophila, TAO1 controls the dynamic interplay between microtubule plus ends and the actin cortex in the regulation of cell morphology [31]. Collectively, these studies showed that TAOKs regulate the dynamics and organization of cytoskeleton proteins, particularly during mitosis.

### 3.5. TAO Kinases Regulate Neuron Development, Neuritogenesis, and Homeostasis

In addition to mitotic cell morphology regulation, TAOKs are involved in neuron development, neuritogenesis, and maintaining a homeostatic neural network via modulating cytoskeleton components. As described above, TAOK1 phosphorylates tau and induces microtubule disassembly. In neurons, TAOK1 activates microtubule affinity-regulating kinase Par1, which then phosphorylates tau and allows the rearrangement of microtubules and development of neurites during neuron differentiation [30] (Figure 3B). TAOK2 acts downstream of Sema3A (Semaphorin 3A) and Nrp1 (Neuropilin 1) and modulates basal dendrite formation and axon elongation during neuron development through activation of the JNK cascade [34]. TAOK1 and 2 are phosphorylated by MST3 (mammalian STE20-like kinase 3), bind with Myosin Va, and relocate to the dendrites to regulate synapse development in neurons [40]. More recently, it was found that TAOK2 phosphorylates the cytoskeletal GTPase Septin7 and participates in maturation of the dendritic spine via stabilization of PSD95 (postsynaptic density protein 95) [41]. Additionally, TAOK2 regulates neurodevelopment and synapse formation via activation of the RhoA signaling pathway [42]. These results indicate that TAOKs regulate the development and differentiation of neurons and formation of synapses through modulating the cytoskeleton components (Figure 3B). Homophilic binding of extracellular arcadlin (rat ortholog of human protocadherin 8/PCDH8) domains activates TAOK2β, a splice variant of TAOK2, which then activates p38 MAPK. The activation of p38 MAPK then positively feeds back to TAOK2β, phosphorylating an essential C-terminal serine 1038 required for endocytosis of the N-cadherin-arcadlin complex at the synapse in hippocampal neurons [43]. It is suggested that PCDH8-mediated N-cadherin endocytosis via TAOK2β signaling is an event within the recovery phase after synaptic stimulation and provides a homeostatic mechanism for maintaining the complexity of the neural network.

### 3.6. Regulation of Inflammation, Immunity, Apoptosis, and other Cellular Pathways by TAOKs

TAOKs are implicated in the regulation of inflammation and immunity. Zhang et al. [44] showed that TAOK1 is a negative regulator of interleukin-17 (IL-17)-mediated signal transduction via preventing the formation of the IL-17 receptor and Act1 (nuclear factor activator 1) complex, thus inhibiting IL-17-associated inflammation and potentially modulating autoimmune progression. TAOK1 was also found to increase the lipopolysaccharide (LPS)-induced production of pro-inflammatory cytokines, including IL-6, TNF-α (tumor necrosis factor-α), and IL12p40, in macrophages [45]. It was found that TAOK1 enhances the LPS-induced activation of ERK1/2 by interacting with TRAF6 (TNF receptor-associated factor 6) and TPL2 (MAP3K8). In this situation, TAOK1 is a positive regulator of the toll-like receptor 4-induced inflammatory responses in macrophages [45]. During positive selection in the spleen, B cell receptor and Notch signaling induces surface expression of ADAM10 in type 1 transitional B cells in a TAOK3-dependent manner, and cells expressing ADAM10 are then committed to marginal zone B in the spleen [46]. As a result, TAOK3 is involved in B cell maturation and fate determination in the spleen. TAOK3 is also reported to be required for canonical T-cell receptor (TCR) signaling through the modulation of SHP-1-dependent LCK (lymphocyte-specific protein tyrosine kinase) inactivation [47]. It was proposed that TAOK3 acts as a binding partner for LCK, diminishing its availability for SHP-1-mediated inactivation. TAOK3 deficiency impairs TCR signaling in human T cells and weakens primary T cell responses [47]. A homozygous missense variant of TAOK2 (c.2098C > T; p.R700C) was found to associate with impaired T cell activation [48], although the underlying mechanism is not known.

TAOKs are involved in apoptosis regulation. Upon treatment of apoptosis-inducing agents including staurosporine and paclitaxel, endogenous TAOK1 and TAOK2 are activated. Activation of TAOK1 in the non-small-cell lung carcinoma cell line H1299 induces cell contraction, membrane blebbing, cleavage of Rho kinase 1 and caspase 3, and activates the JNK pathway for induction of apoptosis [18]. Zihni et al. [19] have also shown that apoptosis-inducing agents stimulate TAOK2-induced JNK and caspase activation and subsequent cleavage and nuclear localization of the N-terminal domain of TAOK2 for apoptosis induction. Interestingly, TAOKs activate caspases, and TAOK1 and 2 have been found to be the substrates of activated caspases [18,19]. TAOKs therefore regulate apoptotic morphological change by reorganizing cytoskeleton proteins via activation of downstream targets including JNK and caspases.

TAOKs are reported to interact with other cytosolic proteins and be involved in additional physiological processes. TAOK2 is found to interact with another MAP3K member, TAK1, and inhibit TAK1-mediated activation of NF-κB by preventing the interaction of TAK1 with IKK (IκB kinase) [49]. Interestingly, the TAK1–TAOK2 complex can still activate JNK, while the TAK1-mediated activation of NF-κB is abolished. This unique regulation is suggested to be a specific cellular response to osmotic stress [49]. Drosophila TAO1 has also been shown to affect the migration of cells during embryonic development [50]. All in all, these results reflect the complexity of TAOK-associated regulation of cell physiology, and the mechanisms we know so far may only represent a small part of the complete story of the TAOK family.

## 4. Role of TAO Kinases in Cancers

Perturbation of kinase signaling resulting from dysregulated expression or activity is often associated with malignant transformation. As described above, studies have shown that TAOKs regulate the activation of several signaling pathways that could be linked to cancer development. However, to date, reports of TAOKs’ correlation with clinical manifestation are few. In cell line studies, TAOKs are reported to be involved in the DNA damage checkpoint of the G2/M transition via p38 MAPK activation. Knockdown of TAOKs not only diminished p38 activation but also impaired the DNA damage response of the G2/M checkpoint [13]. In addition, knockdown of TAOK1 induced various mitotic abnormalities and resulted in chromosome loss in cells [51]. Moreover, TAOK1 and TAOK2 are activated in response to apoptosis-inducing agents and act as regulators of apoptosis by modulating morphological changes including membrane blebbing and the formation of apoptotic bodies via activation of the JNK pathway [18,19]. These studies indicate that dysregulation of TAOKs could be involved in tumorigenesis. In the following sections, we summarize current study results implicating TAOKs in tumor development.

### 4.1. TAO Kinases in Breast Cancer

In a study by Capra et al. [52] using in situ hybridization on tissue microarrays to search for alterations in the expression of serine/threonine kinases in human cancers, TAOK1 was found to be overexpressed in breast cancer tissues compared to normal breast tissues. In a prognostic analysis using a public database (Kaplan–Meier Plotter breast module, probe ID: 220761_s_at; http://kmplot.com/analysis/index.php?p=service&cancer=breast; for details, see [53]), high TAOK3 expression was correlated with poor recurrence-free survival in breast cancer patients who received adjuvant chemotherapy. This study also revealed that TAOK3 enhanced microtubule-targeted drug (paclitaxel, eribulin, and vinorelbine) resistance via the NF-κB signaling pathway in breast cancer cell lines [53] and suggested that disrupting the interaction between TAOK3 and NF-κB signaling may have beneficial therapeutic effects for breast cancer patients treated with anti-microtubule agents. Additionally, in a genomic study, *TAOK1*-*PCGF2* was among the nine fusion genes identified in the breast cancer cell line ZR-75-30 [54].

### 4.2. TAO Kinases in Colorectal Cancer

In the study by Capra et al. [52], TAOK1 was also found to be overexpressed in colorectal cancer tissues compared to normal colon tissues. Conversely, an assay of the kinome profile in colon cancer revealed that TAOK3 is downregulated in adenocarcinoma compared to the normal colon [55]. These results suggest that TAOK1 and TAOK3 have opposite effects in colorectal tumorigenesis, though the underlying mechanism is not clear.

### 4.3. TAO Kinases in Lung Cancer

TAOK1 was found to be overexpressed in lung cancer tissues compared to normal lung tissues in a study using in situ hybridization to search for alterations of serine/threonine kinase expression in cancers [52]. However, in a transcriptome assay of lung adenocarcinoma, TAOK2 was found to be downregulated in tumor tissues compared to the normal lung [56]. Such results may indicate the opposite roles of TAOK1 and TAOK2 in lung tumorigenesis.

### 4.4. TAO Kinases in Pancreatic Cancer

In a pancreatic cancer cell study by Bian et al. [57], expression of TAOK3 was required to support the cancer stem cell-enriching spheroid growth, and knockdown of TAOK3 decreased expression of stem cell traits, spheroid formation, and sensitized cells to gemcitabine treatment. In this study, the authors identified an ITK inhibitor, NCGC00188382, which could inhibit the activity of TAOK3, aurora B kinase, and cyclin-dependent kinase 7 in cancer cells and suppress the stemness traits and growth of pancreatic tumors.

### 4.5. TAO Kinases in Prostate Cancer

The androgen pathway is an important modulator in prostate cancer progression. In the study by Romanuik et al. [58], TAOK3 was found to be an androgen response gene in prostate cancer cells. Furthermore, a later study by Bii et al. [59] confirmed that TAOK3 is a prostate cancer progression-associated gene and expression of TAOK3 can predict the risk of recurrence after androgen deprivation therapy in prostate cancer.

### 4.6. TAO Kinases in Melanoma

In a study by Sharma et al. [60], several kinases including TAOK2 were found to increase ATP uptake in BRAF inhibitor-resistant melanoma cells. In these resistant cells, the activity of TAOK2 is increased and activation of the JNK pathway by TAOK2 is thought to contribute to BRAF inhibitor (vemurafenib) resistance.

### 4.7. TAO Kinases in Larynx Cancer and Leukemia

In the study by Capra et al. [52], TAOK1 was found to be overexpressed in larynx cancer tissues compared to the normal larynx. In acute B lymphoblastic leukemia cells with PAX5 alterations, TAOK1 was found to be a novel fusion partner and the fusion protein PAX5–TAOK1 was proposed to be a competitive inhibitor of wild-type PAX5 for its transactivation activity [61].

**Figure 4:**
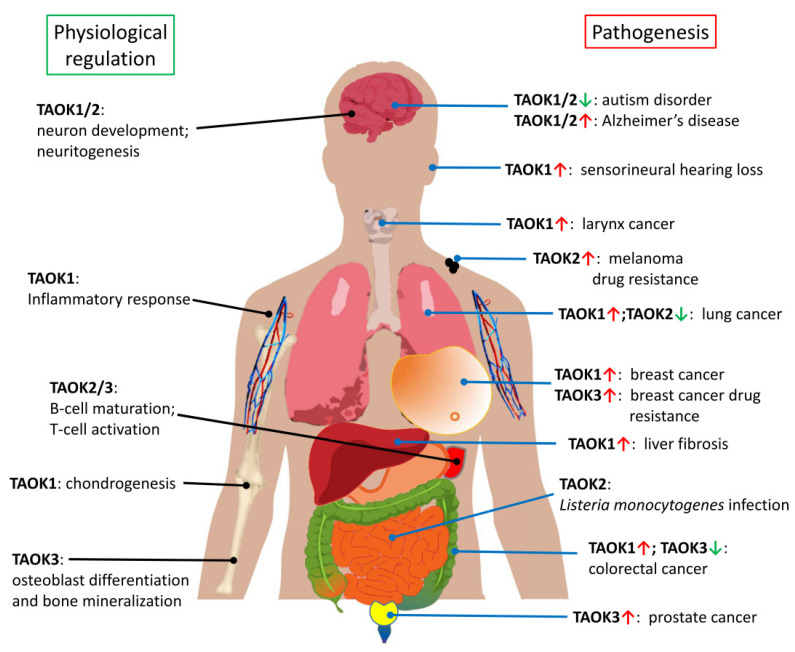
Current knowledge of TAOKs in physiological regulation and pathogenesis. The red “UP” arrows indicate increased expression of TAOK; the green “DOWN” arrows indicate decreased expression of TAOK. This figure contains elements (liver, stomach, and intestine) downloaded from the public domain (see “Acknowledgements” for details).

Currently, most TAOK studies seem to show the tumor-promoting characteristics of TAOKs. However, as aforementioned, TAOKs have also been suggested to act as potential tumor suppressor genes due to their involvement in the Hippo signaling pathway [26] (Figure 2). Consistent with this hypothesis, downregulation of TAOK2 and TAOK3 was noted in lung and colorectal cancer tissues, respectively. The role of each TAOK as a tumor promoter or suppressor under the effect of different cellular contexts or extracellular stimuli remains to be explored (Figure 4).

## 5. TAO Kinases in Cognitive Disorders and Neurodegenerative Diseases

Given that TAOKs are involved in neuron development via modulation of cytoskeleton components, it is not surprising that dysregulation of TAOKs is found to be associated with the development of neural diseases and cognitive disorders. We highlight recent reports in the following sections.

### 5.1. TAO Kinases in Autism Spectrum Disorder

Microdeletion or microduplication of chromosome 16p11.2 has been linked to susceptibility to autism disease [62]. One of the genes from the affected region is TAOK2. TAOK2 downregulation was reported to impair basal dendrite formation while not affecting apical dendrites [34]. This study found that Sema3A and Nrp1 transduce signals via the TAOK2–JNK pathway to regulate basal dendrite development. Low expression of TAOK2 may therefore affect the development of basal dendrites and lead to autism spectrum disorder [34]. Richter et al. [42] subsequently showed that loss of TAOK2 activity causes a reduction in RhoA activation, which then affects F-actin stability in developing neurons and results in reduced brain development and impaired neural connectivity. This study indicates that reduced TAOK2 activity during neural development is associated with autism-related neurodevelopmental and cognitive abnormalities. A study by Dulovic-Mahlow et al. [63] also identified loss-of-function, de novo variants of TAOK1 which correlated with neurodevelopmental disorders. Collectively, these results indicate that abnormal reduction of TAOK1/2 activity during neuron development may result in cognitive abnormalities and lead to the development of autism.

### 5.2. TAO Kinases in Alzheimer’s Disease

As aforementioned, TAOK1 phosphorylates the microtubule-associated protein tau and allows the rearrangement of microtubules and development of neurites during neuron differentiation [30]. However, in Alzheimer’s disease (AD), tau is atypically phosphorylated and aggregates into the characteristic intraneuronal neurofibrillary tangles. Both TAOK1 and TAOK2 have been shown to phosphorylate tau in domains (amino acids 244–368) that are known to regulate the tau–microtubule interactions [30,37] (Figure 3B). The colocalization of catalytically active TAOKs and phosphorylated tau in the AD brain with tangle-bearing neurons has been reported. Such observations suggest that TAOKs participate in the development of dementia and, more specifically, AD by dysregulating tau phosphorylation [32,37].

### 5.3. TAO Kinases in Parkinson’s Disease

In a study of dominant mutation of LRRK2 (leucine-rich repeat kinase 2) in a Parkinson’s disease (PD) model, TAOK3, serine/threonine kinase 3 (STK3), STK24, and STK25 were identified to be novel LRRK2 substrates that may be involved in LRRK2-induced synaptic dysfunction and neurite fragmentation [64]. The kinase cascade from LRRK2 to TAOK, PAR-1, and finally to tau phosphorylation has been proposed as a pathological transformation in tauopathy and axonal pathology in the brains of LRRK2-overexpressing mice and in human PD patients [39,64,65].

### 5.4. TAO Kinases in Cerebral Ischemic Stroke

Although abnormal activity of TAOKs may lead to defects in neuron development, Li et al. [66] have shown that overexpression of TAOK1 ameliorates oxygen glucose deprivation-induced cell injury in neurons and protects rats from induced cerebral ischemic stroke by decreasing pro-inflammatory factors and reducing apoptosis through the PI3K/AKT and MAPK signaling pathways. Taken together, these results support the homeostatic and protective role of TAOKs in the central nervous system under physiological conditions, whereas their dysregulation leads to cognitive disorders and neurodegenerative diseases (Figure 4).

## 6. TAO Kinases in Other Physiological Processes and Diseases

In addition to the aforementioned physiological and pathological roles, TAOKs have been found to regulate additional processes (Figure 4). TAOK3 is reported to function as an upstream activator of the JNK pathway in osteoblasts and its deficiency induces a marked decrease in osteoblast differentiation and defective mineralization [67]. Oxidative stress was found to induce α-SMA (α-smooth muscle actin), PKCα (Protein kinase Cα), and TAOK1 expression during liver fibrogenesis. MicroRNA miR-706 directly inhibits PKCα and TAOK1 expression via binding to their 3′-untranslated regions, thus preventing the epithelial to mesenchymal transition and alleviating hepatic fibrosis [68]. Jing et al. [69] showed that miR-381-3p promoted chondrogenesis in umbilical cord mesenchymal stem cells through direct suppression of TAOK1 and the downstream Hippo signaling pathway, indicating that TAOK1 may regulate chondrogenesis via the Hippo cascade. In an acoustic trauma study by Patel et al. [70], the downregulation of miRNA-183 and upregulation of TAOK1 were observed in noise-traumatized cochlear cells in rats, indicating that the miR-183/TAOK1 pathway is likely to play a role in sensorineural hearing loss.

There are other physiological processes or pathogeneses reported to be TAOK-associated, with mechanisms yet to be defined. In a differentially expressed microarray assay, TAOK1 was found to be highly expressed in patients with coronary artery disease [71]. A missense variant in the kinase domain of TAOK2 (pV244M) was reported in natural killer cell proliferative disorder [72]. A homozygous missense variant of TAOK2 (c.2098C > T; p.R700C) was also found to be associated with impaired T cell activation [48]. Interestingly, TAOK2 was found to be recruited to the internalization vacuole containing intracellular bacteria in *Listeria monocytogenes* infected cells and involved in regulation of vacuolar rupture and cytoplasmic access of these bacteria [73]. Genetic variations in the TAOK3 locus rs795484 were reported to be associated with increased morphine requirement in children of European Caucasian ancestry and with increased acute postoperative pain in both European Caucasian and African American subjects [74,75], although the underlying mechanism is unknown and requires further determination [76]. It has been proposed that TAOK3 may act as a pharmacogene that affects the response to analgesic treatment [77]. Mice with a heterozygous disrupted allele of TAOK3 were resistant to the acute sedative effects of ethanol [22], and those with conditional ablation of TAOK2 were found to recover quickly from ethanol-induced ataxia and consumed increased amounts of ethanol compared with control animals [78]. In a genome-wide methylation analysis, lower methylation of CpG loci within TAOK3 was associated with childhood obesity [79]. TAOK3 was found to be the host kinase that phosphorylates the herpes virus inhibitors methylenecyclopropane nucleosides, instead of viral thymidine kinase [80]. Interestingly, it has also been shown that TAOK3 interacts with the herpes simplex virus structural tegument protein pUL37 [81].

## 7. Current Development of TAOK Inhibitors

Given that TAOKs are involved in many pathological processes and diseases, including apoptosis, inflammation and immune regulation, cancer and drug resistance, autism disorder, and Alzheimer’s disease (Figure 4), the development of specific inhibitors targeting TAOK-related pathways may provide a way to ameliorate their effects on disease progression.

Staurosporine is a broad-range protein kinase inhibitor isolated from *Streptomyces* species. Staurosporine inhibits TAOK2 with an IC50 of 3 μM [82]; however, staurosporine also inhibits a number of other serine/threonine protein kinases with high potency, and this lack of specificity has precluded its use in the clinic. The MST1 inhibitor 9E1 suppresses TAOK2 activity with an IC50 value of 0.3 μmol/L [83]; yet it has the same specificity issue as staurosporine. Recently, two TAOK inhibitors, compounds 43 and 63, were isolated with high specificity to TAOK1, 2, and 3 [33]. Both compounds are ATP-competitive inhibitors of TAOK activity. Compound 43 (N-[2-oxo-2-(1,2,3,4-tetrahydro-naphthalen-1-ylamino)ethyl]biphenyl-4-carboxamide) was found to target and inhibit cancer cells selectively while not affecting non-tumor cells [33]. Compound 43 prolongs the duration of mitosis, reduces the percentage of cells exiting mitosis, and increases mitotic cell death in cancer cells, while non-malignant MCF-10A breast cells continue to proliferate normally [33]. It has also been shown that reducing TAOK expression enhances the sensitivity to γ-radiation in colony survival assays [13], and knockdown of TAOK3 abolishes the drug resistance to microtubule-targeted drugs in breast cancer cells [53]. Therefore, inhibition of TAOK activity by compound 43 may sensitize tumor cells to anticancer treatments. Compound 43 has been demonstrated to decrease tau phosphorylation in murine and human neural cell models of tauopathy. Giacomini et al. [32] have shown that abnormal TAOK activity is present in tauopathies and TAOK inhibition effectively reduces tau phosphorylation on pathological sites. Therefore, compound 43 has the potential to be an effective and specific TAOK inhibitor for further evaluation of its clinical value in cancer, drug resistance, neurodegeneration, and inflammation/autoimmune disorders. In addition, a previously identified ITK inhibitor (NCGC00188382) was shown to inhibit the activity of TAOK3, aurora B kinase, and cyclin-dependent kinase 7 in pancreatic cancer cells and suppress the stemness traits and growth of tumor spheroids [57]. However, given that this ITK inhibitor is a multikinase inhibitor, it is not clear how much of the inhibition is TAOK3-related. Recently, a high-throughput screen of a 200 k compound library identified two additional compounds, SW034538 and SW083688, that showed significant inhibition activity to TAOK2 (IC50 values = 300 nmol/L and 1.3 μmol/L, respectively) [84]. However, the detailed characteristics of these two molecules are currently unknown and remain to be evaluated. The characteristics of currently available TAOK inhibitors are summarized in Table 1.

## 8. Concluding Remarks and Future Perspectives

TAO kinases are members of the MAP kinase cascade whose activators and regulation are only beginning to be uncovered compared to other well-characterized members in this family. In addition to the p38/MAPK and SAPK/JNK cascades, studies have shown that TAOKs are also involved in the Hippo signaling pathway and interact with other cytosolic targets to regulate cellular physiology. However, their upstream activator is largely unknown, and the modulation of TAOK activity under normal physiology and pathogenic conditions remains to be fully deciphered. Although studies are beginning to show that TAOKs regulate immune responses in B and T cells and modulate tissue inflammatory responses via cytokine regulation, further investigation is required to unveil their diverse roles in immune modulation. High expression of TAOK3 in immune cells, and TAOK1/2 in neural cells, may indicate their tissue-specific regulation in the corresponding cells. Based on current knowledge, it seems that certain cellular pathways regulated by TAOK3 are different from those regulated by TAOK1/2. For example, TAOK1 and 2 are involved in cytoskeleton regulation (Figure 3), while the role of TAOK3 in this process is unclear, although a recent study showed that TAOK3 confers resistance to microtubule-targeted drugs in breast cancer cells [53]. Whether this result indicates the involvement of TAOK3 in cytoskeleton modulation in healthy cells requires further investigation. Hence, their tissue-specific expression and diversity in pathway regulations are worth further investigation.

As aforementioned, TAOKs are overexpressed in some tumor tissues while being downregulated in others; however, recent tumorigenic studies of TAOKs were mostly carried out in cell lines and their links to clinical manifestation remain to be fully elucidated. The diversity of TAOKs in modulating the p38/JNK and Hippo pathways is especially worth in-depth investigation, given that they could lead to tumor promoting or inhibiting consequences. Since TAOKs respond to stress conditions, their expression seems to correlate with drug resistance, as demonstrated in breast and melanoma cell studies [53,60]. The underlying mechanisms of drug resistance induced by TAOKs are also of great value for further study. Dysregulation of TAOKs also plays an important role in the tauopathy associated with Alzheimer’s disease and Parkinson’s disease and is implicated in autism-related neurodevelopmental disorders.

The development of TAOK-specific inhibitors seems to be a promising area of research. By targeting their kinase domains, highly specific inhibitors may be selected which do not interfere with other related MAP kinases. However, the efficacy and toxicity of these candidates will need to be evaluated in vivo. Well-planned animal studies are urgently required to evaluate the potency of TAOK inhibitors in disease models before they can proceed to human clinical evaluation. In addition to kinase domain interaction, TAOK2 binds to microtubules and actin through its C-terminal region [7,29]. The development of inhibitors that disrupt the TAOK2 C-terminal interaction may have potential use in the clinic if the interaction is involved in disease pathology. Recently, various kinase inhibitor candidates have been developed by structure-guided, kinase domain virtual screening [85,86]. This is usually achieved by searching a compound that binds to a target through the quantitative structure–activity relationship model derived from existing molecule datasets [85]. Via computer-aided molecular docking, optimal binding modes of ligands for a given binding pocket can be generated, thus facilitating the discovery of specific inhibitors. Since the crystal structures of rat TAOK2 (PDB ID: 1U5Q/1U5R) [87] and human TAOK3 (PDB ID: 6BDN; http://dx.doi.org/10.2210/pdb6bdn/pdb) have been revealed, the application of these methodologies to assist in the development of TAOK-specific inhibitors is achievable and highly anticipated. Given that TAOKs are implicated in many important diseases (summarized in Table 2), the development of a TAOK inhibitor with high specificity and potency could provide a successful treatment for TAOK-associated malignancy and pathogenesis.

## Figures and Tables

**Table 1:** Characteristics of currently available TAOK inhibitors.

Compound Name	IUPAC Name	Cell-Based Inhibition Assay (IC50) (Assay Methodology) *	Kinase Inhibition Assay (IC50) (Assay Methodology) *	Reference
Compound 43	N-[2-oxo-2-(1,2,3,4-tetrahydro-naphthalen-1-ylamino)ethyl]biphenyl-4-carboxamide	NA/showed growth inhibition to SK-BR-3 but not MCF-10A cells	TAOK1: 11 ± 0.44 nmol/L (MBPp) TAOK2: 15 ± 1.63 nmol/L (MBPp) Selectively inhibits TAOK1, 2, 3 while showing low inhibition for other 19 kinases	[33]
Compound 63	N-{3-[(2-{[6-methoxy-1,3-benzothiazol-2yl]amino}-2-oxoethyl)amino]-3-oxo-1-phenylpropyl}benzamide	NA	TAOK1: 19 ± 1.87 nmol/L (MBPp) TAOK2: 39 ± 6.43 nmol/L (MBPp) Selectively inhibits TAOK1, 2, 3 while showing low inhibition for other 19 kinases	[33]
NCGC00188382	*N*-[5-[(3,3-dimethylbutan-2-ylamino)methyl]-1-(2-hydroxy-2-methylpropyl)benzimidazol-2-yl]-5-(1*H*-pyrazol-4-yl)thiophene-2-carboxamide	25 to 300 nmol/L in 24 pancreatic cancer cell lines (FA6 cell: ~25 nmol/L; MDA-Panc-28: ~300 nmol/L) (Cytotoxicity assay)	NA/ showed inhibition to the activity of TAOK3, aurora B kinase, and cyclin-dependent kinase 7 in pancreatic cancer cells	[57]
SW034538	N-(2-((2,5-dimethoxyphenyl)amino)-4’-methyl-[4,5’-bithiazol]-2’-yl)propionamide	NA	TAOK2: 300 nmol/L (MBPp)	[84]
SW083688	N-((2,3-dihydrobenzo[b][1,4]dioxin-2-yl)methyl)-3-(3-ethoxypropyl)-4-oxo-2-thioxo-1,2,3,4-tetrahydroquinazoline-7-carboxamide	NA	TAOK2: 1.3 μmol/L (MBPp)	[84]

* Abbreviations: MBPp: inhibition assay of myelin basic protein phosphorylation; NA: not available.

**Table 2:** The role of TAO kinases in tumorigenesis, inflammation, cognitive/neurodegenerative disorders, and other diseases.

Disease	Sample and Experimental Approach *	Results	Reference
Cancers			
Breast cancer	Biopsy/ISH; cell line/KD and pathway assay	Upregulation of TAOK1 in tumor tissue; TAOK3 enhances microtubule-targeted drug resistance	[52,53]
Colorectal cancer	Biopsy/ISH and kinome profiling	Upregulation of TAOK1 in tumor tissue; downregulation of TAOK3 in adenocarcinoma	[52,55]
Lung cancer	Biopsy/ISH and transcriptome assay	Upregulation of TAOK1 in tumor tissue; downregulation of TAOK2 in tumor tissue	[52,56]
Pancreatic cancer	Cell line/OE and KD, mouse xenograft model	TAOK3 supports the stemness traits and growth of tumor spheroids	[57]
Prostate cancer	Cell line/transcriptome; MGS-PCR	TAOK3 is a prostate cancer progression gene and its expression can predict the risk of recurrence after androgen deprivation therapy	[58,59]
Melanoma	Cell line/activity-based protein profiling	TAOK2 activates JNK and contributes to the BRAF inhibitor (vemurafenib) resistance	[60]
Larynx cancer	Biopsy/ISH	Upregulation of TAOK1 in tumor tissue	[52]
B cell leukemia	Cell line/rolling-circle amplification of cDNA ends	PAX5–TAOK1 fusion protein may be a competitive inhibitor of wild-type PAX5	[61]
Neurodegenerative disease			
Autism	Cell line; mouse model; patient DNA sample/KD and ectopic expression; TAOK2-KO mouse; genotyping	Downregulation of TAOK1/2 activity during neuron development results in cognitive abnormalities and autism	[34,42,62,63]
Alzheimer’s disease	Cell line; mouse model; human biopsy/ OE in cell; IHC; inhibitor assay	TAOK1/2 dysregulate tau phosphorylation and participate in the development of dementia and AD	[32,37]
Parkinson’s disease	Cell line/protein array assay	TAOK3 is a novel LRRK2 substrate and involved in LRRK2-induced PD	[64]
Cerebral ischemic stroke	Mouse model/induced cerebral ischemic stroke	TAOK1 ameliorates induced cerebral ischemic stroke by decreasing pro-inflammatory factors and reducing apoptosis	[66]
Inflammation			
IL-17-associated	Cell line/KD and OE; TAOK1 KO-mouse model	TAOK1 inhibits IL-17-mediated signal transduction and inflammation	[44]
LPS-induced	KO-mouse model of TAOK1 in myeloid cells	TAOK1 enhances LPS-induced activation of ERK1/2 and positively regulates the TLR4-induced inflammatory response	[45]
Other diseases			
Liver fibrosis	Mouse model of liver fibrosis	miR-706 inhibits PKCα and TAOK1 expression, thus prevents liver fibrosis	[68]
*Listeria* infection	Bacteria and cell line/siRNA microscopy screening	TAOK2 regulates vacuolar rupture and cytoplasmic access of *Listeria*	[73]
Coronary artery disease	Expression database analysis	TAOK1 highly expressed in patients with coronary artery disease	[71]
Sensorineural hearing loss	Noise-traumatized rat model/ miRNA expression analysis	Downregulation of miRNA-183 and upregulation of TAOK1 may be involved in sensorineural hearing loss	[70]

* Abbreviations: AD: Alzheimer’s disease; EMT: epithelial–mesenchymal transition; IHC: immunohistochemistry; ISH: in situ hybridization; KD: knockdown; KO: knockout; LPS: lipopolysaccharide; MGS-PCR: modified genomic sequencing PCR; OE: overexpression; PD: Parkinson’s disease; TLR4: toll-like receptor 4.
